# Writing System Modulates the Association between Sensitivity to Acoustic Cues in Music and Reading Ability: Evidence from Chinese–English Bilingual Children

**DOI:** 10.3389/fpsyg.2017.01965

**Published:** 2017-11-09

**Authors:** Juan Zhang, Yaxuan Meng, Chenggang Wu, Danny Q. Zhou

**Affiliations:** Faculty of Education, University of Macau, Macau, China

**Keywords:** music, language, reading, writing system, Chinese

## Abstract

Music and language share many attributes and a large body of evidence shows that sensitivity to acoustic cues in music is positively related to language development and even subsequent reading acquisition. However, such association was mainly found in alphabetic languages. What remains unclear is whether sensitivity to acoustic cues in music is associated with reading in Chinese, a morphosyllabic language. The present study aimed to answer this question by measuring music (i.e., musical metric perception and pitch discrimination), language (i.e., phonological awareness, lexical tone sensitivity), and reading abilities (i.e., word recognition) among 54 third-grade Chinese–English bilingual children. After controlling for age and non-verbal intelligence, we found that both musical metric perception and pitch discrimination accounted for unique variance of Chinese phonological awareness while pitch discrimination rather than musical metric perception predicted Chinese lexical tone sensitivity. More importantly, neither musical metric perception nor pitch discrimination was associated with Chinese reading. As for English, musical metric perception and pitch discrimination were correlated with both English phonological awareness and English reading. Furthermore, sensitivity to acoustic cues in music was associated with English reading through the mediation of English phonological awareness. The current findings indicate that the association between sensitivity to acoustic cues in music and reading may be modulated by writing systems. In Chinese, the mapping between orthography and phonology is not as transparent as in alphabetic languages such as English. Thus, this opaque mapping may alter the auditory perceptual sensitivity in music to Chinese reading.

## Introduction

Reading is an essential ability for growing children (Lonigan, 2007; Tong and McBride-Chang, 2010). Children initially learn to speak and then to read. As they are exposed to print increasingly, reading skills that are the bases of learning other knowledge improve accordingly. In other words, children usually develop from “learn to read” to “read to learn.” Research conducted in the past decades demonstrated that music training (e.g., rhythm skills, singing, instrument playing, etc.) could enhance reading development based on a similar mechanism underlying music, language, and reading processing (Tierney and Kraus, 2013).

### Music and Language

Thus far, much evidence has shown that music perception ability (e.g., sensitivity to acoustic cues in music) is closely related to language skills, such as phonological awareness. Anvari et al. (2002), for example, examined the associations between music perception and phonological awareness. They found that for 4- and 5-year-old children, music perception was strongly correlated with phonological awareness (Anvari et al., 2002). Huss et al. (2011) further examined the associations between music perception and phonological awareness among typically developing children and dyslexic children. The results revealed that music metrical sensitivity predicted phonological awareness not only for normal children, but also for dyslexic children. In addition to correlational studies, experimental data also corroborated that music training could enhance phonological skills (Register, 2004; Gromko, 2005; Moreno et al., 2009, 2011; Degé and Schwarzer, 2011; Cogo-Moreira et al., 2013; Moritz et al., 2013; Thomson et al., 2013). For instance, Degé and Schwarzer (2011) investigated whether 20-week music training could improve phonological awareness as phonological training program did. The phonological training program targeted to improve children’s rhyming, phoneme recognition, and syllable recognition skills. Forty-one preschool children were randomly assigned to music training group, phonological training group, and sports training group (i.e., control group). Each group was daily trained 10 min for 20 consecutive weeks. After the training, enhancement of phonological awareness was found in both music training and phonological training groups, supporting that music training had similar facilitation effect on phonological awareness as phonological training did and there existed a shared learning mechanism of language and music for preliterate children. In one recent meta-analysis, Gordon et al. (2015) synthesized previous studies and conducted a meta-analysis that showed a small but significant enhancement of music on phonological awareness.

Thus far, several accounts have been proposed to explain the positive association between music perception and language development (Goswami, 2011; Slevc, 2012; Patel, 2014). For example, as argued by Patel (2003), music perception skills can be transferred to language skills, and vice versa based on the fact that although music and language have different syntactic representations (e.g., chords versus words), music and language share the same neural resources for syntactic integration (Patel, 2003). In addition, music perception requires individuals to be sensitive to acoustic cues in music, such as pitch and rhythm. In music, the temporal and spectral units represent rhythm and pitch respectively, and in language, prosody can vary through rhythm and pitch at the same time. Therefore, sensitivity to acoustic cues in music (e.g., pitch and rhythm) is also presumed to be related to language processing (Chung et al., 2017).

### Music and Reading

Since oral language skills pave the foundation for reading development (Nichd Early Child Care Research Network, 2005; Miller et al., 2006) and music training (e.g., musical improvisation, composition, and interpretation) can enhance the development of phonological awareness (Cogo-Moreira et al., 2013; Flaugnacco et al., 2015) and syntax knowledge (Patel et al., 1998) that broadly belong to oral language skills, it is possible that music training is also positively associated with children’s reading abilities (Kraus et al., 2014).

In recent years, increasing evidence has demonstrated that the reason why music training is facilitating for reading development is that music aptitude is positively linked to reading development. Here, music aptitude (i.e., audition) refers to the abilities to hear or feel music (Gordon, 1986). For instance, Strait et al. (2011) explored music aptitude and reading ability by recording brainstem responses to speech among typically developing children aged from 3 to 8 years. The structural equation modeling analysis showed that music aptitude predicted around 38% variance of reading ability by affecting auditory memory and neural sensitivity to speech regulation. Moreover, in a longitudinal study, Dellatolas et al. (2009) investigated the association between music rhythmic production and reading ability among 695 French children. All children were initially tested on music rhythm production as well as working memory and attention measurements when they were kindergarteners (Time 1) and then their reading abilities were measured when they were second graders (Time 2). The researchers found that rhythm production assessed in kindergartens could uniquely predict reading ability in the second grade with other cognitive abilities (i.e., visual working memory, attention) statistically controlled. Furthermore, the association between rhythmic production and reading was not only observed for typically developing children, but also found for dyslexic children (Dellatolas et al., 2009).

In fact, music aptitude was primarily tested with pitch and rhythm perception (Gordon, 1965, but see Dellatolas et al., 2009, for rhythm production). Recently, a strong relation between pitch perception and reading was identified (Zhang and McBride-Chang, 2014) and relation between rhythm perception and reading was also observed (Huss et al., 2011). These studies implied a possible association between sensitivity to acoustic cues in music (e.g., pitch and rhythm) and reading (Tierney and Kraus, 2013). The reason why sensitivity to acoustic cues in music could be related to reading is that, according to related reading models such as temporal sampling framework (Goswami, 2011), auditory processing lays the foundation for language and reading development (Zhang and McBride-Chang, 2010, 2014; Wang et al., 2012; Antoniou et al., 2015; Chung et al., 2017) and auditory processing is also vividly involved in music processing (Gordon, 1965), sensitivity to acoustic cues in music is consequently assumed to be positively associated with reading.

### Music and Chinese Reading

Despite the intriguing findings, previous studies investigating the role of music on language and reading development have mainly focused on alphabetic languages. Chinese, a morphosyllabic language, has captured increasing attention of researchers recently (Mok and Zuo, 2012; Wu et al., 2015; Tang et al., 2016). Two factors necessitate the investigation of the relationship between sensitivity to acoustic cues in music and reading abilities in Chinese. The first factor is that Chinese is a tonal language and the heavy reliance on lexical tone perception to develop Chinese language and reading skills permits a positive transfer from sensitivity to acoustic cues in music especially pitch discrimination to language and subsequent reading skills. More specifically, Chinese lexical tones, which are realized in pitch variations, influence Chinese word recognition. For example, the syllable /wu*/* can represent different meanings when combined with different lexical tones, with */*wu1*/* meaning *house* 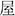, /wu2*/* meaning *no* 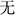, /wu3*/* meaning *five* 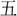, and /wu4*/* meaning *frog* 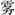. Despite that Chinese speakers are immersed in tonal communication environment, sensitivity to acoustic cues in music is still associated with their lexical tone processing skills. For example, Tang et al. (2016) employed the oddball paradigm to examine whether music aptitude could facilitate tone perception for Chinese speakers. The results revealed that after age and intelligence were controlled, musicians showed enhanced mismatch negativity (MMN) and faster discrimination performance than non-musicians. The second reason necessitating the investigation of music and Chinese reading is that Chinese writing system is of unique characteristics that could tremendously impact children’s reading development (Shu et al., 2003; McBride, 2015). One prominent property is that phonology and orthography in Chinese is mapped at the syllable level such that Chinese is phonologically opaque. Therefore, Chinese children cannot read aloud Chinese characters through grapheme–phoneme conversion as children speaking alphabetic languages do (Yeung et al., 2011). This uniqueness may modulate the positive relation between sensitivity to acoustic cues in music and Chinese reading ability. Thus far, there are few investigations that aim to examine this claim. One exception was that recently Lu and Greenwald (2016) examined the relations among music aptitude, Chinese reading, and working memory in Chinese adults. Participants were required to read music notation or visual Chinese words and decide whether the stimuli pairs (i.e., homophonic Chinese words, or music notations) were same or not in tone or pitch (i.e., tones for homophonic words, or pitch for music notations). It was found that adults who received extensive music training and were better at music notation reading also performed better at discriminating the tonal information of words than those with lower performance on music notation reading. The intriguing results indicate that music perception was positively related to processing of lexical tone information in reading, but it is still unclear whether the same findings hold true for children since Lu and Greenwald only examined Chinese adults. Moreover, musicians in that study (Lu and Greenwald, 2016) had been trained in music for over 16 years and taken formal Chinese education for over 15 years, suggesting that they were skilled Chinese readers and music experts. It is widely believed that skilled reading of Chinese as against alphabetic languages, such as English, is “ultimately more similar than different,” but the process of learning to read Chinese for children is systematically different from English (McBride, 2016). Hence, for developing children, it remains unclear whether sensitivity to acoustic cues in music is positively related to Chinese reading skills or not.

Therefore, the current study aimed to extend previous investigations of sensitivity to acoustic cues in music in relation to language and reading abilities in alphabetic-language speakers to Chinese–English bilingual children. In general, the present study concentrated on two research questions. The first question was how sensitivity to acoustic cues in music was associated with Chinese language and reading abilities. More specifically, it was hypothesized that sensitivity to acoustic cues in music was related to Chinese phonological awareness based on previous studies (Tierney and Kraus, 2013; Gordon et al., 2015). In addition, sensitivity to acoustic cues in music might be also associated with Chinese tone sensitivity according to previous research comparing musicians and those without music training (Tang et al., 2016). However, there might be no significant association between sensitivity to acoustic cues in music and Chinese reading abilities due to the unique characteristic of Chinese writing system. The characteristic is that the mapping between phonology and orthography is opaque at the syllable level instead of at the phoneme level and phonology is activated only when the activation of the orthography of a word reaches certain threshold instead of through the direct grapheme–phoneme conversion (Perfetti and Harris, 2013). The second research question was how sensitivity to acoustic cues in music was associated with children’s language and reading skills in their second language (i.e., English). Majority of the previous studies explored music enhancement on language and reading ability in children’s first language, so it is unclear whether such transfer effect could be observed in their second language (i.e., English). Based on previous literature, it was hypothesized that music skills were positively associated with both English phonological awareness and English reading because mounting evidence indicated that music training could enhance English phonological awareness and lead to gains in English reading (Tierney and Kraus, 2013; Flaugnacco et al., 2015; Gordon et al., 2015). More importantly, since phonological awareness is a strong predictor for English reading development (Melby-Lervåg et al., 2012) and it could be enhanced by music program training (Degé and Schwarzer, 2011), it was hypothesized that sensitivity to acoustic cues in music was linked to English reading through the mediation of phonological awareness.

## Materials and Methods

### Participants

Fifty-four third-grade primary school students (32 boys, 22 girls) aged between 8 and 10 years (*M* = 8.57 years, *SD* = 0.67) were recruited from two elementary schools in Macau. The first language and the medium of instruction in schools for all the students were Cantonese. Meanwhile, English was taught as a second language when they were around 3 years old, suggesting that they were sequential Chinese–English bilinguals. Informed and written consent forms were obtained from the parents or guardians of these students prior to the formal test. In addition, the protocol for the tests was approved by the Institutional Review Board in the University of Macau.

The whole study was divided into three parts. The first part aimed to measure the language and reading abilities through phonological awareness (Chinese, English), lexical tone sensitivity (Chinese), and word reading (Chinese, English). The second part assessed children’s sensitivity to acoustic cues in music through musical metric perception task and pitch discrimination task. In the third part, children’s non-verbal intelligence was measured.

### Procedure

Trained female Cantonese-speaking research assistants administered all the tasks to each child. The whole study lasted for about 2 h and participants could have a rest whenever necessary during the testing. The tests were conducted counterbalanced in order among participants.

### Measurements

#### Language and Reading Abilities

##### Chinese word reading

The test was adopted from the reading and writing subtest of the Hong Kong Test of Specific Learning Difficulties (HKT-SpLD) (Ho et al., 2000) and was used to tap Chinese word reading skills of children. The test consisted of 150 two-character words (e.g., 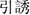 /jan5 jau5/ meaning *allure*) and participants were asked to read the words aloud one by one. The difficulty level of these items increased from the easiest to the most difficult. Children would get a score of 1 if they read the word correctly and were awarded a score of 0 if they didn’t know the word or mispronounced the word. The test ended when the child was unable to give correct answers for 15 consecutive items. The maximum possible score in this task was 150.

##### English word reading

This task was adopted from one previous study to tap English reading abilities for children from second to fifth grade in Hong Kong (Tong and McBride-Chang, 2010). There were 60 English words in total and all the words were divided into six sets with increasing difficulty levels. The instruction for this task was quite similar to that of the Chinese word reading task and children were instructed to read the words aloud one by one. The test would stop when children could not give four correct responses within one difficulty level. Participants were awarded a score of 1 for each correct answer and the possible maximum score in this task was 60.

##### Chinese lexical tone sensitivity

**FIGURE 1:**
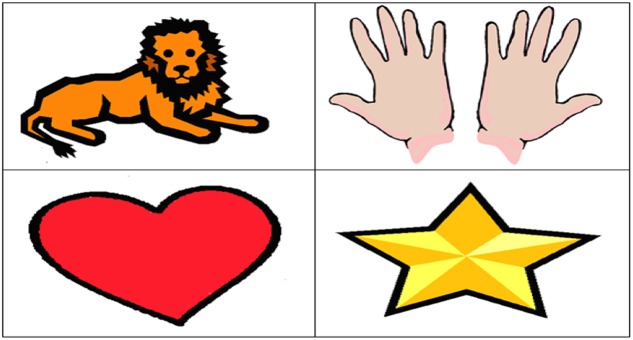
Example picture for Chinese lexical tone sensitivity task.

The Chinese lexical tone sensitivity task was used in previous studies to assess children’s abilities to discriminate the tones of Chinese words (Cheung et al., 2009; Tong and McBride-Chang, 2010; Zhang and McBride-Chang, 2014). In each trial of this task, children were shown four pictures with each describing a one-syllable word. One of the four words differed from the other three in lexical tone and children were instructed to pick the picture with the odd tone out. As shown in **Figure 1**, for example, participants were first asked to speak out the four words /si1/ meaning *lion*, /sau2/ meaning *hand*, /sam1/ meaning *heart*, and /seng1/ meaning *star*. Then they should point out the one with the odd tone. The correct answer should be the second picture (/sau2/ meaning *hand*). There were 24 items in total and the possible maximum score was 24.

##### Phonological awareness (Chinese)

There were 51 items in total and these items were divided into six sets (K3 to P5) according to the difficulty levels. The test began with a designated level according to children’s grade (P3) and was guided by a ceiling and a basal rule. Specifically, there were seven items in P3 level and the test would go back to the preceding easier level if the correctly answered items by the child were fewer than two in this level and stepped further to the more difficult level if more than three items were correctly responded. The test stopped if the child answered only two or three items correctly in one certain level. Two subtests, including syllable deletion and phoneme deletion were included. A sample item for the syllable deletion subtest would be: 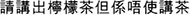 (meaning *please speak out lemon tea without saying tea*). The answer should be 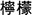 (meaning *lemon*). For the phoneme deletion subtest, an example could be: 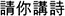 (si1) 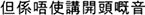 (meaning *please say* /si1/ *without the first phoneme*) and the correct answer should be /i/. The maximum score in this task was 51.

##### Phonological awareness (English)

The test consisted of two parts, targeting to tap both syllable and phoneme awareness. In the syllable deletion subtest, participants were asked to say a multi-syllable English word without one certain syllable. For example, the participants were instructed to say /foot/ and the correct answer should be /ball/. In the phoneme deletion task, participants were asked to say one single-syllable word without one certain phoneme. For example, they were instructed to speak out /hug/ without /h/ and the correct answer was /ug/. Participants would get a score of 1 for each correct answer and a score of 0 for wrong answers. The test stopped once the children incorrectly answered four consecutive items. The possible maximum score in this task was 36. This task was used in one previous study to tap Chinese children’s phonological awareness in English (e.g., Zhang and McBride-Chang, 2014).

#### Sensitivity to Acoustic Cues in Music

Sensitivity to acoustic cues in music was assessed through two tasks including musical metric perception task and pitch discrimination task, both of which were programmed using E-prime 2.0.

##### Musical metric perception task

In each trial of this task, two series of musical notes were presented sequentially and each musical line comprised of six musical notes whose pitch was G (392 Hz). All the 20 trials were in 4/4 times and half of these trials were identical and the other half were different by prolonging the duration of one certain musical note among the musical line. Specifically, following Huss et al. (2011), metrical structure was changed by adding 100 ms to the music notes (short duration change) or by prolonging 166 ms to the musical notes (long duration change). Participants were instructed to press “f” for the same rhythms and press “j” for the different rhythms. Each musical line lasted for 5000 ms and the inter-stimuli interval was 500 ms. The materials were delivered in a random order. There were 20 trials in total and the possible maximum score was 20. This task was used in previous studies to assess meter sensitivity (e.g., Huss et al., 2011).

##### Pitch-discrimination task

This task took the same format of the abovementioned musical metric perception task. There were 36 trials in total and each trial consisted of one pair of musical arrangements. Half of the trials were identical while the other half were different by changing the pitch height of one certain note among the musical line. All sound files were created by the software Sibelius Version 7 and each of the six notes was altered by being moved either higher or lower in the diatonic scale of C major to create a combination of different arrangements. The maximum score in this task was 36. Similar task was used in recent studies to tap pitch discrimination of Chinese children (Jiang et al., 2015).

#### Non-verbal Intelligence Test

Raven’s Standard Progressive Matrices (Set A + Set B) were used to tap children’s non-verbal reasoning skills. Each set had 12 items and there were 24 items in total. In each trial, children were shown a visual geometric design with a missing part. They should pick one from six pictures that could best fit in the missing part. The possible maximum score in this task was 24. Same task was used in recent studies to tap children’s non-verbal intelligence (Zhang and McBride-Chang, 2014).

## Results

The descriptive information for all the variables is shown in **Table 1**. **Table 2** shows the zero-order correlations among all the variables and their partial correlations with age and non-verbal intelligence statistically controlled.

**Table 1:** Descriptive analysis for all tasks.

	Minimum	Maximum	Mean	*SD*
Raven test	10	23	19.09	2.56
Chinese word recognition	34	140	98.61	19.65
English word reading	0	23	5.74	6.31
Chinese lexical tone	6	18	10.30	3.15
Phonological awareness (Chinese)	24	51	37.00	7.86
Phonological awareness (English)	5	64	20.44	9.32
Pitch discrimination	16	35	24.54	4.40
Musical metric perception	6	19	12.06	2.99

*The score in Raven’s IQ test was obtained by adding the raw scores from Set A and Set B together*.

**Table 2:** Correlations and partial correlations among variables.

	1	2	3	4	5	6	7	8	9
(1) Age	-								
(2) Non-verbal IQ	0.18	-							
(3) Chinese word reading	-0.07	0.56	-	0.34*	0.36**	0.42**	0.22	0.17	0.13
(4) English word reading	-0.11	0.12	0.35**	-	0.27	0.25	0.53**	0.29*	0.31*
(5) Chinese lexical tone	-0.002	0.35*	0.36**	0.30*	-	0.17	-0.03	0.48**	0.22
(6) Chinese phonological awareness	0.03	0.14	0.42**	0.26	0.21	-	0.37**	0.42**	0.50**
(7) English phonological awareness	0.07	0.21	0.22	0.53**	0.04	0.39**	-	0.40**	0.43**
(8) Pitch discrimination	0.17	0.15	0.16	0.27*	0.48**	0.42**	0.42**	-	0.37**
(9) Musical metric perception	-0.12	-0.05	0.14	0.31*	0.19	0.49**	0.41**	0.33*	-

*^∗^p < 0.05, two tailed*; ^∗∗^p* < 0.01, two tailed. The left bottom shows the zero-order correlations among the variables, and the right top shows their partial correlations when controlling for age and non-verbal intelligence*.

As shown in the **Table 2**, zero-order correlation analysis revealed that Chinese lexical tone sensitivity was significantly associated with pitch discrimination (*r* = 0.48) rather than with musical metric perception (*r* = 0.19). Both pitch discrimination and musical metric perception were significantly correlated with Chinese phonological awareness (*r* = 0.42 and 0.49, respectively) while neither of them was significantly associated with Chinese word reading (*r* = 0.16 and 0.14, respectively). Chinese word reading was found to correlate with Chinese tonal awareness (*r* = 0.36) and Chinese phonological awareness (*r* = 0.42).

To further explore the relationships among different variables and answer the first research question: how sensitivity to acoustic cues in music was associated with Chinese language and reading abilities (i.e., Chinese lexical tone sensitivity and Chinese phonological awareness), hierarchical regressions were conducted.

Firstly, the contribution of pitch discrimination to the Chinese lexical tone sensitivity was tested and the results are shown in **Table 3**. Age and non-verbal intelligence were entered in step 1 as control variables and musical metric perception was further controlled in Step 2. It was found that pitch discrimination (Step 3) could uniquely explain 16% variance of Chinese lexical tone sensitivity (β = 0.44, *t* = 3.40, *p* < 0.01) when age, non-verbal intelligence, and musical metric perception were statistically controlled, with the whole model accounting for 27% variance. Secondly, the relationship between sensitivity to acoustic cues in music and Chinese phonological awareness was also examined (see **Table 4**). Age and non-verbal IQ were first controlled in Step 1. When musical metric perception was further controlled in Step 2, pitch discrimination could explain 5% unique variance of Chinese phonological awareness (β = 0.27, *t* = 2.09, *p* < 0.01). Similarly, when pitch discrimination was further controlled in the second step, musical metric perception could account for 13% unique variance of Chinese phonological awareness (β = 0.40, *t* = 3.17, *p* < 0.01). Therefore, both pitch discrimination and musical metric perception could uniquely account for Chinese phonological awareness.

**Table 3:** Hierarchical regression predicting Chinese lexical tone.

Step	Variables	β	*t*	*R^2^*	Δ*R^2^*
1	Age	-0.07	-0.50	0.09	0.09^∗^
	Non-verbal IQ	0.36	2.68^∗^		
2	Musical metric perception	0.20	1.56	0.11	0.02^∗^
3	Pitch discrimination	0.44	3.40^∗∗^	0.27	0.16^∗∗^

*^∗^p < 0.05*; ^∗∗^p* < 0.01*.

**Table 4:** Hierarchical regression predicting Chinese phonological awareness.

Step	Variables	β	*t*	*R^2^*	Δ*R^2^*
1	Age	-0.001	-0.005	-0.02	-0.02
	Non-verbal IQ	0.14	1.00		
2	Musical metric perception	0.50	4.09**	0.22	0.24**
3	Pitch discrimination	0.27	2.09*	0.27	0.05*
2	Pitch discrimination	0.42	3.22**	0.14	0.16**
3	Musical metric perception	0.40	3.17**	0.27	0.13**

^∗^*p < 0.05; ^∗∗^*p* < 0.01*.

The second question concerned how sensitivity to acoustic cues in music was associated with children’s language and reading skills in their second language English. As shown in **Table 2**, both pitch discrimination and musical metric perception were correlated with English phonological awareness (*r* = 0.42 and 0.41, respectively). To explore the unique contributions of pitch discrimination and musical metric perception to English phonological awareness, hierarchical regression analyses were conducted and results are shown in **Table 5**. Age and IQ were entered in Step 1 as control variables. When musical metric perception was further controlled in Step 2, pitch discrimination explained 5% unique variance of English phonological awareness (β = 0.28, *t* = 2.14, *p* < 0.05). Meanwhile, musical metric perception could account for 8% unique variance of English phonological awareness (β = 0.33, *t* = 2.49, *p* < 0.05) when pitch discrimination was further controlled in Step 2. The results showed that both pitch discrimination and musical metric perception could independently explain English phonological awareness. As for the roles of music and languages skills on English reading, as shown in **Table 2**, both pitch discrimination and musical metric perception were also significantly correlated with English word reading (*r* = 0.27 and 0.31, respectively). Meanwhile, English phonological awareness was a significant correlate of English word reading (*r* = 0.53). To explore the role of English phonological awareness in the association between sensitivity to acoustic cues in music and English reading, hierarchical regressions were conducted. As shown in **Table 6**, with age and non-verbal intelligence controlled in Step 1 and both of the pitch discrimination and musical metric perception further controlled in Step 2, English phonological awareness could still account for 15.2% unique variance of English word reading (β = 0.47, *t* = 3.29, *p* < 0.01). However, when English phonological awareness was controlled in Step 2, pitch discrimination and musical metric perception together failed to explain unique variance of English word reading. The results indicated that sensitivity to acoustic cues in music might have an impact on English reading through the English phonological awareness.

**Table 5:** Hierarchical regression predicting English phonological awareness.

Step	Variables	β	*t*	*R^2^*	Δ*R^2^*
1	Age	0.04	0.26	0.01	0.01
	Non-verbal IQ	0.20	1.44		
2	Musical metric perception	0.43	3.40^∗∗^	0.18	0.17^∗^
3	Pitch discrimination	0.28	2.14^∗^	0.23	0.05^∗^
2	Pitch discrimination	0.40	3.12^∗∗^	0.15	0.14^∗^
3	Musical metric perception	0.33	2.49^∗^	0.23	0.08^∗^

*^∗^p < 0.05*; ^∗∗^p* < 0.01*.

**Table 6:** Hierarchical regression predicting English word reading.

Step	Variables	β	*t*	*R^2^*	Δ*R^2^*
1	Age	-0.13	-0.95	-0.006	-0.006
	Non-verbal IQ	0.14	1.03		
2	Musical metric perception	0.24	1.66	0.09	0.10^∗^
	Pitch discrimination	0.20	1.38		
3	English phonological awareness	0.47	3.29^∗∗^	0.24	0.15^∗^
2	English phonological awareness	0.53	4.39^∗∗^	0.26	0.26^∗∗^
3	Musical metric perception	0.08	0.61	0.24	-0.02
	Pitch discrimination	0.07	0.49		

*^∗^p < 0.05*; ^∗∗^p* < 0.01*.

**FIGURE 2:**
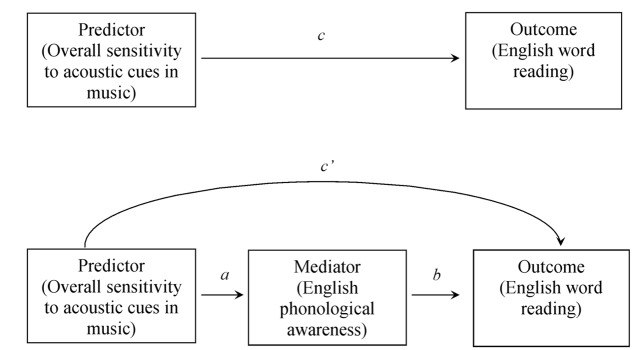
Input model: English phonological awareness mediating overall sensitivity to acoustic cues in music and English word reading.

To further test the hypothesis that sensitivity to acoustic cues in music was linked to English reading through the mediation of phonological awareness, the mean accuracies of the two music tasks were averaged to index the overall sensitivity to acoustic cues in music and additional hierarchical regressions were conducted to directly examine the mediating effect of English phonological awareness. Technically, four conditions should be met for a mediation effect (Baron and Kenny, 1986). Firstly, the overall sensitivity to the acoustic cues in music (predictor) should be significantly regressed on English word reading (outcome) (*c′*). Secondly, the English phonological awareness (mediator) should be significantly associated with English word reading (outcome) (*b*). Thirdly, the predictor should be significantly related to the mediator (*a*). Finally, the effect of the predictor on the outcome should be decreased (*c′ < c:* partial mediation) or become non-significant (full mediation) when the mediator is included (**Figure 2**). As shown in **Table 7**, the relationships between outcome and predictor (β = 0.36, *t* = 2.78, *p* < 0.05), as well as the association between the outcome and the mediator (β = 0.53, *t* = 4.48, *p* < 0.01) were both significant. The relationship between the predictor and mediator was also significant (β = 0.51, *t* = 4.22, *p* < 0.01). When adding the mediator to the regression equation, the relation between the predictor and outcome became non-significant (β = 0.13, *t* = 0.91, *p* = 0.37), while the association between the mediator and outcome was still significant (β = 0.47, *t* = 3.40, *p* < 0.01). Thus, the above results indicated that the relationship between the predictor and outcome was fully mediated by the mediator. That is, English phonological awareness served a mediating function between sensitivity to acoustic cues in music and English word reading (see **Figure 2**).

**Table 7:** Hierarchical regression examining the mediating effect of English phonological awareness.

	Condition 1		Condition 2		Condition 3		Condition 4	
	β	*t*	β	*t*	β	*t*	β	*t*
Combined sensitivity to acoustic cues in music	0.36	2.78^∗^	–	–	0.51	4.22^∗∗^	0.13	0.91
English phonological awareness	–	–	0.53	4.48^∗∗^	–	–	0.47	3.40^∗∗^

*^∗^p < 0.05*; ^∗∗^p* < 0.01*.

## Discussion

There were two central research questions in this study. The first question was how sensitivity to acoustic cues in music was associated with language skills (e.g., phonological awareness) and reading skills in Chinese. This question was derived from two considerations. The first consideration was that it was necessary to separate language and reading, because (1) language is generally learnt earlier than reading and serves as a foundation for reading development (Nichd Early Child Care Research Network, 2005); (2) reading involves mapping the orthography to phonology and semantics to identify words and therefore it requires more efforts to acquire (Perfetti and Harris, 2013). The second consideration is that Chinese has at least two prominent characteristics. One characteristic is that Chinese is a tonal language, and there is evidence that suggests sensitivity to acoustic cues in music can enhance tone perception, even for Chinese speakers who are immersed in tonal communication on daily basis (Tang et al., 2016). Additionally, tone perception was a significant correlate of Chinese children’s reading development (McBride-Chang et al., 2008). It is possible that music training can also facilitate Chinese reading via enhancing lexical tone sensitivity due to the fact that in music, the temporal and spectral units represent the rhythm and pitch, while prosodic information can be described through rhythm and pitch in language as well. The other characteristic is that the script of Chinese is different from those of alphabetic languages such as English because the mapping between orthography and phonology in Chinese is at the syllable level rather than at the phoneme level as in English or other alphabetic languages. That is, Chinese is much more phonologically opaque compared to alphabetic languages. The above two considerations prompted us to examine the first research question about the relationship among sensitivity to acoustic cues in music and Chinese language and reading abilities among Chinese-speaking children.

In the current study, we found that Chinese phonological awareness and lexical tonal sensitivity were correlated with Chinese reading ability, which replicated previous findings that phonological awareness and tone sensitivity are strong predictors for reading development in Chinese (Shu et al., 2008; Song et al., 2016). Additionally, pitch discrimination was found to be associated with Chinese phonological awareness in the present study, which is consistent with existing literature. For example, Zhang and McBride-Chang (2014) found that individual differences in pitch discrimination were significantly correlated with Chinese phonological awareness among second and third graders in Hong Kong. Similarly, Goswami et al. (2011) also found that pitch discrimination explained 17.5% variance of Chinese phonological awareness among Taiwan children. Our finding of the association between musical metric perception and Chinese phonological awareness was also analogous to the finding that musical metric perception was a strong predictor for phonological awareness (Huss et al., 2011). It was also worth noting that there was a strong association between Chinese tone sensitivity and pitch discrimination. It was plausible that children in the current study were immersed in Cantonese (i.e., tonal language) speaking environment that facilitated their pitch sensitivity. Moreover, the present study found that pitch discrimination uniquely predicted Chinese tone sensitivity, which is in line with the evidence that there was a significant correlation between pitch discrimination and lexical tone perception (Zhang and McBride-Chang, 2014).

However, one prominent finding in the current study was that there was no significant association between sensitivity to acoustic cues in music and Chinese reading. This result was in contrast with previous evidence that music experience positively predicted reading skills and reading development in alphabetic languages such as English (Forgeard et al., 2008; Dellatolas et al., 2009; Moreno et al., 2009, 2011; Strait et al., 2011; Cogo-Moreira et al., 2013; Thomson et al., 2013). This inconsistency may be resulted from the uniqueness of Chinese writing system. Learning to read requires the understanding of how writing system encodes its oral language (Perfetti and Harris, 2013). According to the orthographic depth hypothesis (Katz and Frost, 1992), mapping between orthography and phonology varies across writing systems. More specifically, the mapping from print to phonology in Chinese is more opaque than in alphabetic languages. Chinese is a morphosyllablic language, in which a morpheme, the smallest meaning unit in oral language, generally corresponds to a syllable phonologically and a character in print. Chinese learners cannot explicitly decompose the syllable into phonemes in order to read Chinese characters/words successfully (Yeung et al., 2011). Seidenberg (2011) moved beyond orthographic depth hypothesis and argued that the “division of labor” differs across writing systems. Specifically, within a complicated and multicomponent system (e.g., orthography, phonology, semantics), one needs efficiently divides the labor to solve a task, such as word recognition (Seidenberg, 2011). Since mapping from print to phonology in Chinese is less transparent than in alphabetic languages, a more efficient way for Chinese reading is to rely more on the route of orthography to semantics than the route from orthography to phonology (Yang et al., 2008, 2009). Therefore, Chinese children may not rely on phonological processing and auditory processing as much as English-speaking children do (Tan et al., 2005). Even if Chinese phonological awareness strongly predicted Chinese reading development as revealed in a recent meta-analysis (Song et al., 2016), the effect size of Chinese phonological awareness for predicting Chinese reading (*r* = 0.36) was much smaller than English phonological awareness (*r* = 0.57) for predicting English reading (Melby-Lervåg et al., 2012; Song et al., 2016). In fact, morphological awareness, which refers to the awareness of morpheme structure of words and the ability to manipulate the morpheme structure plays a more pivotal role for Chinese children’s reading development than phonological awareness does (McBride-Chang et al., 2003; Shu et al., 2006; McBride, 2016). Some study even suggests that Chinese morphological awareness is a stronger predictor (β = 0.509) for Chinese reading than other language skills such as phonological awareness (β = 0.192) and vocabulary (β = 0.200) (Shu et al., 2006). However, more recently, Lu and Greenwald (2016) found that musician adults outperformed their counterparts in reading Chinese words, which implies a positive transfer from music training to reading ability. Actually, Lu and Greenwald (2016) recruited musicians with more than 16-year music training. It was conceivable that with continuous and intensive music training, a possible positive transfer from music aptitude to Chinese reading might be observed. Our finding of no association between sensitivity to acoustic cues in music and Chinese reading does not necessarily preclude the potential facilitation effect of music training on Chinese reading, provided that music training is extensive and lasts for a long time. Additionally, the relationship between sensitivity to acoustic cues in music and Chinese reading might be identified because of the speech features in Chinese that is a tonal language (Tang et al., 2016). Therefore, another possibility that no association between sensitivity to acoustic cues in music and Chinese reading was that the musical metric perception task (Huss et al., 2011) failed to capture the speech features in Chinese in the present study. In this task, Huss et al. (2011) manipulated “duration” of the auditory notes and consequently altered the beat distribution and one supra-segmental trait in English, namely stress, is strongly linked to the sound intensity and duration. Thus, it was natural to observe that English language and reading were associated with musical metric perception while Chinese reading was not, because Chinese is not a stress language. Future study might consider modifying the musical metric perception task that allowed more variation in pitch for Chinese children and a possible association between sensitivity to acoustic cues in music and Chinese reading could probably be identified.

The second research question in the present study was how sensitivity to acoustic cues in music was associated with children’s language and reading abilities in their second language (i.e., English) given that most of the previous studies mainly attempted to investigate such associations in children’s first language. In the current study, we investigated sensitivity to acoustic cues in music in relation to phonological awareness and reading in English as a second language. The results first showed that sensitivity to acoustic cues in music was closely related to English phonological awareness, supporting many previous investigations (Anvari et al., 2002; Degé and Schwarzer, 2011; Moreno et al., 2011; Goswami et al., 2013). By separately testing the roles of pitch discrimination and musical metric perception of children, regression analyses further revealed that both pitch discrimination and musical metric perception uniquely and independently predicted phonological awareness (Kraus et al., 2014). The results were consistent with previous correlational (Anvari et al., 2002; Magne et al., 2006; Huss et al., 2011; Goswami et al., 2013) and experimental (Degé and Schwarzer, 2011; Moritz et al., 2013) studies that showed associations among music training, speech perception, and phonological skills. More importantly, sensitivity to acoustic cues in music was positively correlated with English reading in this study. Furthermore, sensitivity to acoustic cues in music was linked to English reading through the mediation of phonological awareness. The result was in line with the notion that auditory sensitivity was indirectly associated with English reading by affecting phonological processing especially phonological awareness (McBride-Chang, 1996). The finding is also consistent with a large body of research which indicated that musical training could facilitate phonological awareness (Degé and Schwarzer, 2011) and reading (Proverbio et al., 2013) in alphabetic languages. The mediation role of phonological awareness explained why music training can serve as an effective intervention for children who are struggling with language and reading, at least for alphabetic languages (Tierney and Kraus, 2013).

Several limitations in current study warrant further discussion. Firstly, this study took a correlational design, so it was hard to establish a causal relationship between sensitivity to acoustic cues in music and language and reading development. Future experimental investigations may further explore the enhancement effect of sensitivity to acoustic cues in music on language and reading abilities by adding a control group and an experimental intervention. Secondly, other English language skills (e.g., prosodic skills in English such as lexical stress sensitivity) were not explored in the present study. Future study might investigate whether English stress sensitivity is associated with English word reading and their relations with sensitivity to acoustic cues in music. Thirdly, the current study only tested a group of children at one time point, failing to reveal how language and reading abilities develop with the increasing exposure to music over time. A longitudinal study could be conducted to investigate the dynamic relations among music experience, and language and reading development. In this way, a developmental trajectory of the relations will be understood. Finally, we only tested the single word reading. Future studies might consider investigating relationship between music and reading by examining other reading abilities, such as reading fluency and reading comprehension (Gordon et al., 2015).

Despite these limitations, the present examination captured theoretical significance in at least the following aspects. The first one is that our results extend previous studies (Foxton et al., 2003; Wang et al., 2012; Antoniou et al., 2015; Chung et al., 2017) by indicating that Chinese–English bilingual children can benefit from sensitivity to acoustic cues in music to facilitate their language and reading skills in their second language (i.e., English). More crucially, the second contribution is that even if sensitivity to acoustic cues in music was correlated with Chinese tone perception and Chinese phonological awareness, we found no significance association between sensitivity to acoustic cues in music and Chinese reading. This noticeable result informs that positive relation between sensitivity to acoustic cues in music and Chinese reading development may not be as strong as English reading development, because (1) there is an opaque mapping between orthography and phonology in Chinese writing system, (2) Chinese children who are learning to read do not necessarily crack the syllable into phonemes to read Chinese fluently (Yeung et al., 2011; Perfetti and Harris, 2013), and (3) morphological awareness, instead of phonological awareness, is the strongest predictor for Chinese children’s reading development (Shu et al., 2006).

## Author Contributions

JZ developed the research idea. JZ and YM conducted the experiments. YM performed data analyses. JZ, CW, and YM wrote the manuscript. DZ created the stimuli for the pith discrimination task.

## Conflict of Interest Statement

The authors declare that the research was conducted in the absence of any commercial or financial relationships that could be construed as a potential conflict of interest.
